# Reappraisal of Metformin Efficacy in the Treatment of Type 2 Diabetes: A Meta-Analysis of Randomised Controlled Trials

**DOI:** 10.1371/journal.pmed.1001204

**Published:** 2012-04-10

**Authors:** Rémy Boussageon, Irène Supper, Theodora Bejan-Angoulvant, Nadir Kellou, Michel Cucherat, Jean-Pierre Boissel, Behrouz Kassai, Alain Moreau, François Gueyffier, Catherine Cornu

**Affiliations:** 1 Department of General Medicine, Université Claude Bernard Lyon 1, Lyon, France; 2 Service de Pharmacologie Clinique, Centre Hospitalier Régional et Universitaire de Tours, France; 3 UMR 7292, CNRS, Université François Rabelais, Tours, France; 4 UMR 5558, CNRS, Laboratoire de Biométrie et Biologie Évolutive, Villeurbanne, France; 5 Université Claude Bernard Lyon 1, Lyon, France; 6 Clinical Investigation Centre, INSERM CIC201, Lyon, France; 7 Department of Clinical Pharmacology, Hospices Civils de Lyon, Lyon, France; Lund University Diabetes Centre, Sweden

## Abstract

**Background:**

The UK Prospective Diabetes Study showed that metformin decreases mortality compared to diet alone in overweight patients with type 2 diabetes mellitus. Since then, it has been the first-line treatment in overweight patients with type 2 diabetes. However, metformin-sulphonylurea bitherapy may increase mortality.

**Methods and Findings:**

This meta-analysis of randomised controlled trials evaluated metformin efficacy (in studies of metformin versus diet alone, versus placebo, and versus no treatment; metformin as an add-on therapy; and metformin withdrawal) against cardiovascular morbidity or mortality in patients with type 2 diabetes. We searched Medline, Embase, and the Cochrane database. Primary end points were all-cause mortality and cardiovascular death. Secondary end points included all myocardial infarctions, all strokes, congestive heart failure, peripheral vascular disease, leg amputations, and microvascular complications. Thirteen randomised controlled trials (13,110 patients) were retrieved; 9,560 patients were given metformin, and 3,550 patients were given conventional treatment or placebo. Metformin did not significantly affect the primary outcomes all-cause mortality, risk ratio (RR) = 0.99 (95% CI: 0.75 to 1.31), and cardiovascular mortality, RR = 1.05 (95% CI: 0.67 to 1.64). The secondary outcomes were also unaffected by metformin treatment: all myocardial infarctions, RR = 0.90 (95% CI: 0.74 to 1.09); all strokes, RR = 0.76 (95% CI: 0.51 to 1.14); heart failure, RR = 1.03 (95% CI: 0.67 to 1.59); peripheral vascular disease, RR = 0.90 (95% CI: 0.46 to 1.78); leg amputations, RR = 1.04 (95% CI: 0.44 to 2.44); and microvascular complications, RR = 0.83 (95% CI: 0.59 to 1.17). For all-cause mortality and cardiovascular mortality, there was significant heterogeneity when including the UK Prospective Diabetes Study subgroups (*I* ^2^ = 41% and 59%). There was significant interaction with sulphonylurea as a concomitant treatment for myocardial infarction (*p* = 0.10 and 0.02, respectively).

**Conclusions:**

Although metformin is considered the gold standard, its benefit/risk ratio remains uncertain. We cannot exclude a 25% reduction or a 31% increase in all-cause mortality. We cannot exclude a 33% reduction or a 64% increase in cardiovascular mortality. Further studies are needed to clarify this situation.

*Please see later in the article for the Editors' Summary*

## Abstract 2

Catherine Cornu and colleagues performed a meta-analysis of randomised controlled trials of metformin efficacy on cardiovascular morbidity or mortality in patients with type 2 diabetes and showed that although metformin is considered the gold standard, its benefit/risk ratio remains uncertain.

## Editors' Summary

**Background:**

Worldwide, more than 350 million people have diabetes, and this number is increasing rapidly. Diabetes is characterized by dangerous amounts of sugar (glucose) in the blood. Blood sugar levels are normally controlled by insulin, a hormone produced by the pancreas. In people with type 2 diabetes (the most common form of diabetes), blood sugar control fails because the fat and muscle cells that usually respond to insulin by removing excess sugar from the blood become less responsive to insulin. Type 2 diabetes can be controlled with diet and exercise and with antidiabetic pills, each of which works in a different way to maintain a healthy blood sugar level. Metformin, for example, stops the liver making glucose and increases the body's response to insulin, whereas sulfonylureas help the pancreas make more insulin. The long-term complications of diabetes, which include an increased risk of cardiovascular problems such as heart disease and stroke, reduce the life expectancy of people with diabetes by about ten years compared to people without diabetes.

**Why Was This Study Done?:**

In 1998, a large randomized clinical trial called the UK Prospective Diabetes Study (UKPDS 34) reported that metformin in combination with dietary control reduced all-cause mortality in overweight patients with type 2 diabetes when compared to dietary control alone. Specifically, the risk of death from any cause among patients taking metformin was about a third lower than the risk of death among patients not taking metformin—a risk ratio (RR) of 0.64. This reduction in risk was significant (that is, it was unlikely to have occurred by chance) because its 95% confidence interval (95% CI; there is a 95% chance that the “true” RR lies within this interval) of 0.45–0.91 did not overlap 1.0. Given this finding, metformin is now recommended as the first-line treatment for type 2 diabetes. However, UKPDS 34 also reported an increase in death in non-overweight patients who took metformin plus sulfonylurea compared to those who took sulfonylurea alone (RR: 1.60; 95% CI: 1.02–2.52), a result considered non-significant by the UKPDS 34 researchers and largely ignored ever since. So do the benefits of metformin outweigh its risks? In this meta-analysis, the researchers re-evaluate the risk-to-benefit balance of metformin in the treatment of patients with type 2 diabetes. A meta-analysis is a statistical method that combines the results of several studies.

**What Did the Researchers Do and Find?:**

The researchers identified 13 randomized controlled trials that evaluated the effect of metformin on cardiovascular morbidity (illness) and mortality in patients with type 2 diabetes. More than 13,000 patients participated in these studies, three-quarters of whom received metformin and a quarter of whom received other treatments or a placebo. Compared to other treatments, metformin treatment had no effect on the risk of all-cause mortality (RR: 0.99; 95% CI: 0.75–1.31) or cardiovascular mortality (RR: 1.05; 95% CI: 0.67–1.64), the primary end points of this study. However, the results of the individual trials varied more than would be expected by chance (“heterogeneity”). Exclusion of the UKPDS 34 trial from the meta-analysis had no effect on the estimated risk ratio for all-cause mortality or cardiovascular deaths, but the heterogeneity disappeared. Finally, metformin treatment had no significant effect on the risk of cardiovascular conditions such as heart attacks, strokes, and heart failure; there was no heterogeneity among the trials for these secondary end points.

**What Do These Findings Mean?:**

These findings show no evidence that metformin has any beneficial effect on all-cause mortality, on cardiovascular mortality, or on cardiovascular morbidity among patients with type 2 diabetes. These findings must be cautiously interpreted because only a few randomized controlled trials were included in this study, and only a few patients died or developed any cardiovascular illnesses. Importantly, however, from these findings, it is impossible to exclude beyond reasonable doubt the possibility that metformin causes up to a 25% reduction or a 31% increase in all-cause mortality. Similarly, these findings cannot exclude the possibility that metformin causes up to a 33% reduction or a 64% increase in cardiovascular mortality. Given that a large number of patients take metformin for many years as a first-line treatment for diabetes, further studies are urgently needed to clarify this situation.

**Additional Information:**

Please access these web sites via the online version of this summary at http://dx.doi.org/10.1371/journal.pmed.1001204.

- The International Diabetes Federation provides information about all aspects of diabetes
- The US National Diabetes Information Clearinghouse provides information about diabetes for patients, health-care professionals, and the general public, including detailed information on diabetes medicines (in English and Spanish)
- The UK National Health Service Choices web site provides information for patients and carers about type 2 diabetes and includes peoples stories about diabetes
- The charity Diabetes UK also provides detailed information for patients and carers, including information on diabetes medications, and has a further selection of stories from people with diabetes
- MedlinePlus provides links to further resources and advice about diabetes and about diabetes medicines; it also provides information about metformin (in English and Spanish)
- The charity Healthtalkonline has interviews with people about their experiences of diabetes and of controlling diabetes with oral medications

## Introduction

Type 2 diabetes mellitus (T2DM), is a major health problem because of its cardiovascular complications and economic costs [1]. Epidemiological evidence indicates that T2DM is an independent risk factor for cardiovascular diseases (CVDs). The rate of CVDs is approximately two times higher in diabetic patients than non-diabetic patients [2]. Since publication of the results of the UK Prospective Diabetes Study (UKPDS 34) in 1998 [3], metformin, a biguanid glucose-lowering agent, has been recommended as the first-line treatment by international guidelines [4],[5]. When compared with diet alone, metformin showed a reduction of all-cause mortality in overweight patients (risk ratio [RR] = 0.64; 95% CI: 0.45 to 0.91 [3]). In the same study, non-overweight patients were randomised to receive various glucose-lowering treatments, and some took either metformin and sulphonylurea or sulphonylurea alone. An increase of overall mortality (RR = 1.60; 95% CI: 1.02 to 2.52) was observed in the metformin add-on sulphonylurea group when compared with sulphonylureas alone. The authors attributed this disturbing result to chance. The authors of recently published Cochrane systematic reviews on metformin efficacy did not include this result in their analyses [6]. Their conclusion, based on the results of the overweight patient group, is that metformin reduces overall and cardiovascular mortality. Selvin et al. [7] and Bennett et al. [8] also did not include the results of non-overweight group, even though they mentioned this subgroup. They concluded that “treatment with metformin hydrochloride was associated with a decreased risk of cardiovascular mortality (pooled OR, 0.74; 95% CI, 0.62–0.89) compared with any other oral diabetes agent or placebo” [7]. Lamanna et al. [9] integrated both subgroups, but included non-diabetic patients as well as patients with HIV or polycystic ovary syndrome. They also did not include safety studies as Rachmani et al. [10] and COSMIC [11] did. They concluded that “it is likely that metformin monotherapy is associated with improved survival (MH-OR: 0.801[0.625–1.024], p = 0.076). However, concomitant use with sulphonylurea was associated with reduced survival (MH-OR: 1.432[1.068–1.918], p = 0.016)” [9].

Phenformin, a drug belonging to the same biguanid family as metformin, was withdrawn from the market after an increased cardiovascular mortality rate was observed in the University Group Diabetes Program study [12].

Our aim was to review all available evidence to evaluate the risk-to-benefit balance of metformin in T2DM patients based on cardiovascular morbidity and mortality using a systematic review and meta-analysis of controlled trials.

## Methods

### Data Sources

Studies were identified by searching Medline, Embase, and the Cochrane database of systematic reviews (1 January 1950 through 31 July 2010) with the following key words: type 2 diabetes, diabetes mellitus; macrovascular; cardiovascular or coronary diseases, stroke, peripheral vascular disease; microvascular; retinopathy; neuropathy; nephropathy; and metformin. No language restrictions were applied. Reference lists of published meta-analyses were reviewed.

### Study Selection

Included studies were randomised controlled trials that evaluated metformin effects in T2DM patients on cardiovascular morbidity or mortality as primary outcomes, secondary outcomes, or adverse events. We included studies comparing metformin to diet alone, placebo, or no treatment, as well as studies of metformin as an add-on therapy, i.e., a comparison of metformin versus no treatment combined with another treatment, and studies of metformin withdrawal. We did not include active-control metformin monotherapy studies.

Two investigators (R. B. and I. S.) independently reviewed the identified abstracts or manuscripts to determine which studies were eligible for inclusion in the meta-analysis.

### Quality Assessment

The quality of selected articles was assessed by two independent investigators (R. B. and I. S.) using the Jadad score [13].

### End Points

Two reviewers (R. B. and I. S.), independently and in duplicate, extracted numerical data for all the outcomes of interest from the included trials.

Primary end points were all-cause mortality and cardiovascular death. Secondary end points included: all myocardial infarctions (fatal and non-fatal), all strokes (fatal and non-fatal), congestive heart failure, peripheral vascular disease, leg amputations, and microvascular complications. End-point definitions referred to what was reported in the originally published papers. End points were not available for all studies included in this meta-analysis. Therefore, our evaluation was not always based on the overall studied population.

### Statistical Analysis

For each trial, RRs and 95% CIs were calculated from the number of events in each group using a fixed-effects model. Summary data for each end point were obtained by pooling the RRs across studies. Statistical heterogeneity across trials was assessed with the χ^2^ statistic (*p*<0.1) and the *I* ^2^ statistic [14]. The *I* ^2^ statistic measures the proportion of overall variation that is attributable to between-study heterogeneity. The heterogeneity test was considered statistically significant if the *p*-value was under 0.1. Heterogeneity was considered high if the *I^2^* was above 50%. Tau^2^ was calculated in order to determine the size and clinical relevance of heterogeneity when detected by the previous calculations [15]. A random-effects model was used when the heterogeneity test was statistically significant. Sensitivity analyses and an interaction test were performed based on (a) the Jadad score (≤3 versus >3) and (b) sulphonylurea as an add-on treatment (absent versus present).

Statistical analyses were performed according to the intention-to-treat principle. All *p*-values were two-sided (*p*<0.05). Analyses were performed using Revman software, version 5 (http://ims.cochrane.org/revman).

## Results

**Figure 1:**
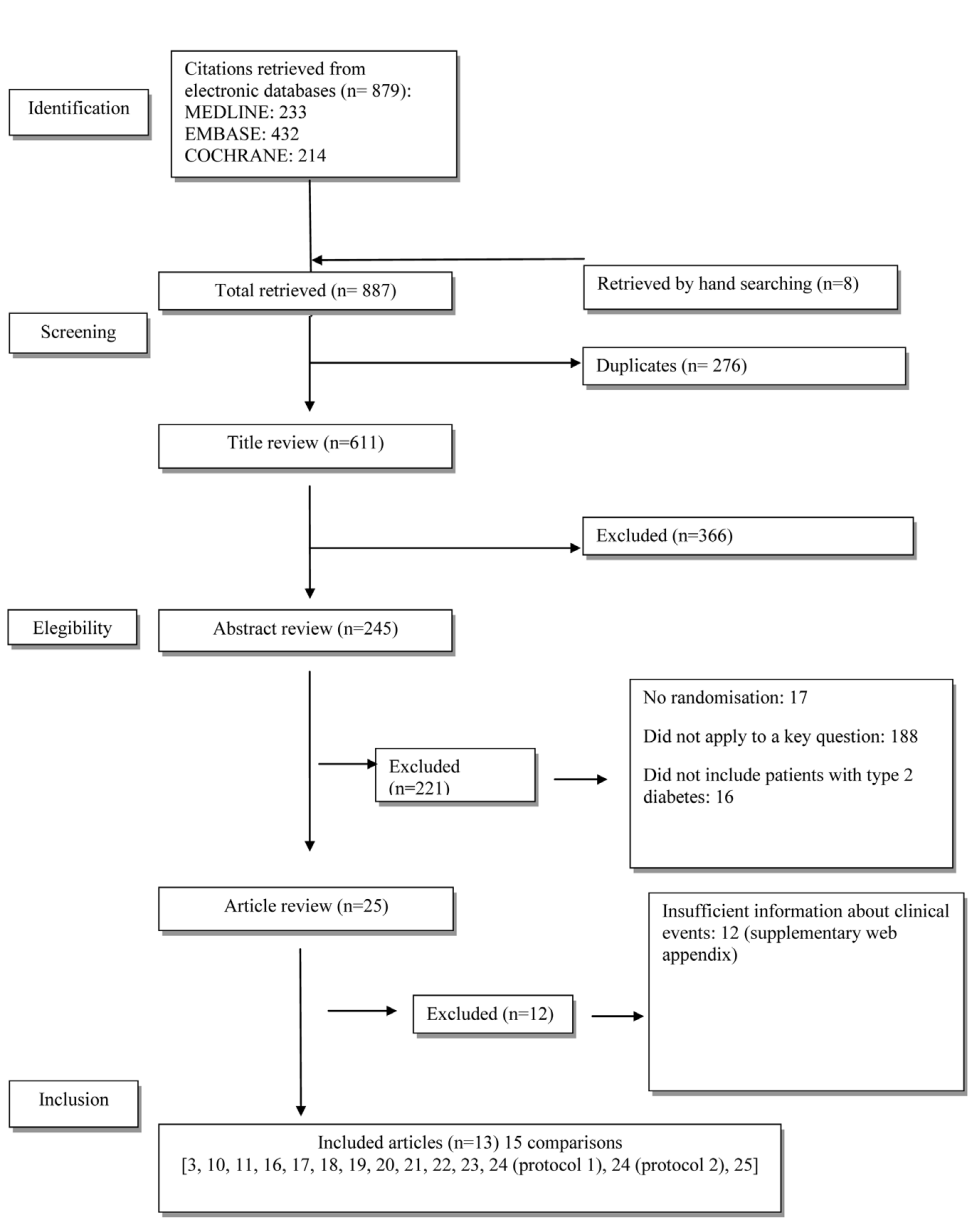
Flow chart.

The flow diagram of study selection is shown in Figure 1. Overall, 25 trials met the inclusion criteria. Twelve trials were excluded because they did not report sufficient information about clinical events (see Text S2). Only four had a clinical event as the primary outcome: one double-blind controlled trial, Hyperinsulinemia: The Outcome of its Metabolic Effects (HOME) [16], and three open trials, UKPDS 34 [3], Rachmani et al. [10], and COSMIC [11]. Nine trials had clinical events as adverse events [17]–[25]. In five studies, metformin was given as an add-on to sulphonylurea [3],[18],[22]–[24], and in two studies, as an add-on to insulin [16],[20]; two studies were versus diet [3],[17], and two were versus usual care [10],[11].

The baseline characteristics of the selected studies are summarised in Table 1.

**Table 1:** Characteristics of Studies or Subgroups Included in the Meta-Analysis.

Study	Trial Characteristics							Patient Characteristics				
	Jadad Score Double-Blind (Yes/N0)	Participants *n* (Metaformin/Control)	Treatments	Follow-Up (Months)	Inclusion Criteria		Primary End Point	Males (Percent)	Age (Years)	BMI (kg/m^2^)	Duration of Diabetes (Years)	Initial HbA1c (Percent)
					HbA1c/FPG/Current Treatment	Overweight						
Teupe and Bergis [17]	3 N	100 (50/50)	M/diet	24	FPG 120–180 mmol/l	NS	Metabolic control	40	53.7	NA	NA	9
Hermann et al. [18]	4 Y	106 (72/34)	M+SU/Pbo+SU	6	FPG≥6.7 mmol/l	NS	Glycaemia	63	60	NA	4	6.8
DeFronzo and Goodman, Protocol 1 [24]	4 Y	289 (143/146)	M/Pbo	29	Diet alone	Y	FPG	74	53	30	6	8.3
DeFronzo and Goodman, Protocol 2 [24]	4 Y	422 (213/209)	M+SU/SU	29	FPG>7.8 mmol/l	120%–170% of ideal	FPG	85	55	29	8	8.8
UKPDS 34(a) [3]	3 N	753 (342/411)	M/diet	128	FPG 6.1–15.0 mmol/l	Y	Clinical events	47	53	31.8	<1	7.1
UKPDS 34(b) [3]	3 N	537 (268/269	M+SU/SU	78	FPG 6.1–15.0 mmol/l	Y+N	Clinical events	60	58	29.7	<1	7.5
Chiasson et al. [25]	4 Y	166 (83/83)	M/Pbo	36	HbA1c 7.2%–9.5%	NS	HbA1c	75	57	31.1	5.1	8.1
Horton et al. [19]	4 Y	350 (178/172)	M/Pbo	6	HbA1c 6.8%–11%	BMI 20–35	HbA1c	64	58.5	NA	NA	8.3
Hermann et al. [20]	4 Y	35 (16/19)	M+I/Pbo+I	12	HBA1c>reference+2%	Y	Glycaemia	54	57.5	NA	NA	8.9
Blonde et al. [23]	4 Y	486 (322/164)	Association M+SU/SU	4	HbA1c≥7.4	BMI≤40	HbA1c	57	56	30	7	9.6
Rachmani et al. [10]	Withdrawal trial, 3 N	393 (195/198)	M+UC/UC	48	NS	BMI 24–40	Clinical events	51	64	28.5	14.5	8.6
Hällsten et al. [21]	4 Y	29 (15/14)	M/Pbo	6	Newly diagnosed/diet-treated	NS	Muscle glucose uptake	66	58	NA	NA	6.6
Garber et al. [22]	4 Y	322 (171/151)	M+SU/Pbo+SU	4	HbA1c 7%–12%	BMI 20–40	HbA1c	44	55	31	NA	8.7
Cryer et al. (COSMIC) [11]	3 N	8,732 (7,227/1,505)	M+UC/UC	12	Suboptimally controlled	NS	Clinical events	50	57.7	30	4.8	NA
Kooy et al. (HOME) [16]	4 Y	390 (196/194)	M+I/Pbo+I	51	NS	NS	Clinical events	45.6	61.5	30	13	7.9

FPG, fasting plasma glucose; I, insulinotherapy; M, metformin; N, no; NA, not available; NS, not specified; Pbo, placebo; SU, sulphonylureas; UC, usual care; Y, yes.

UKPDS 34 was divided into two parts. UKPDS 34(a) evaluated metformin plus diet versus diet alone, and UKPDS 34(b) evaluated metformin plus sulphonylurea versus sulphonylurea alone.

The present meta-analysis included 13,110 patients (Table 1). Among them, 50% were men; their mean age (range) was 57.7 (53–64) y; baseline mean body mass index (BMI) (range) was 30 (28.5–31.8) kg/cm^2^. The mean (range) duration of diabetes was 4.8 (0–14.5) y. In total, 9,560 patients were randomised to receive metformin, and 3,550 to receive the conventional or placebo treatment.

**Figure 2:**
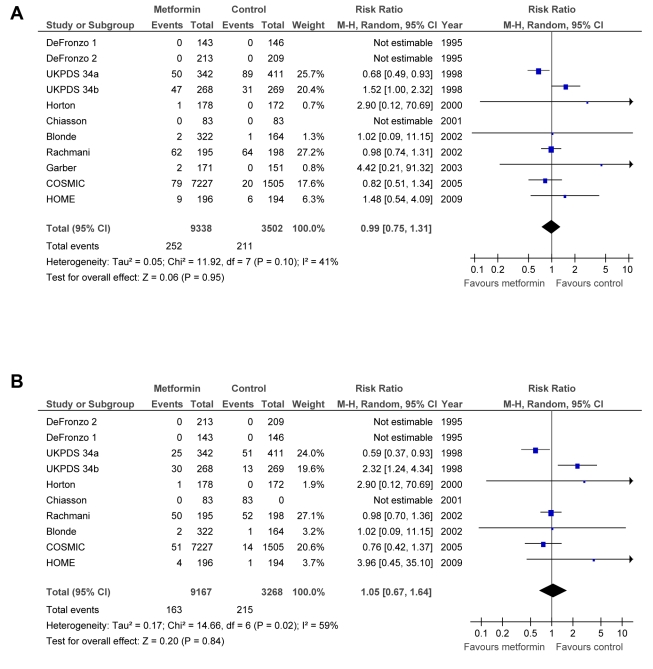
Forest plot for primary end points. (A) All-cause mortality. (B) Cardiovascular mortality. df, degrees of freedom; M-H, Mantel–Haenszel odds ratio method.

The effect of metformin on mortality and macrovascular complications is summarised in Figure 2.

### Primary End Points

Metformin did not significantly affect the primary end points: all-cause mortality (RR = 0.99; 95% CI: 0.75 to 1.31) or cardiovascular deaths (RR = 1.05; 95% CI: 0.67 to 1.64) (Figure 2). There was significant heterogeneity between trials for all-cause mortality (*p* = 0.10, Tau^2^ = 0.05, *I* ^2^ = 41%) and cardiovascular deaths (*p* = 0.02, Tau^2^ = 0.17, *I* ^2^ = 59%). The results did not change after restricting the analysis to trials with a Jadad score >3 or trials with clinical events as outcomes. The analysis of trials where metformin plus sulphonylurea was compared to sulphonylurea alone (see Text S1) shows a significant increase in all-cause mortality, RR = 1.53 (95% CI: 1.02 to 2.31), and in cardiovascular deaths, RR = 2.20 (95% CI: 1.20 to 4.03). UKPDS 34(a) represents most of the weight of this analysis.

After excluding UKPDS 34, the estimated RR for all-cause mortality (RR = 0.98; 95% CI: 0.77 to 1.24) and cardiovascular deaths (RR = 0.95; 95% CI: 0.72 to 1.26) did not change (plot not shown), but no heterogeneity was detected for all-cause mortality (*p* = 0.77, Tau^2^ = 0.00, *I* ^2^ = 0%) or cardiovascular deaths (*p* = 0.61; Tau^2^ = 0.00, *I* ^2^ = 0%). After excluding UKPDS 34(a), UKPDS 34(b), or both, the results remained not significant (plot not shown), and heterogeneity disappeared.

### Secondary End Points

**Figure 3:**
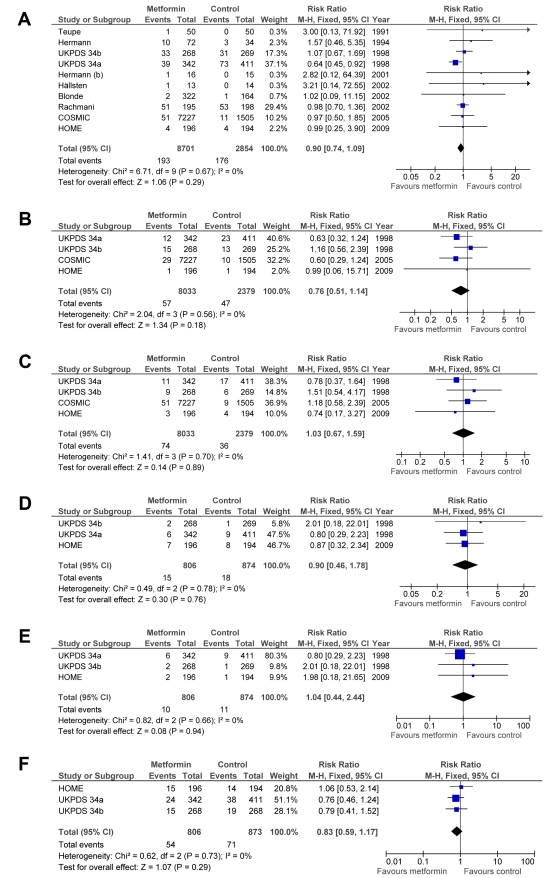
Forest plot for secondary end points. (A) All myocardial infarctions. (B) All strokes. (C) Heart failure. (D) Peripheral vascular events. (E) Amputation. (F) Microvascular complications. df, degrees of freedom; M-H, Mantel–Haenszel odds ratio method.

The rates of all myocardial infarctions (RR = 0.90; 95% CI: 0.74 to 1.09), all strokes (RR = 0.76; 95% CI: 0.51 to 1.14), heart failure (RR = 1.03; 95% CI: 0.67 to 1.59), peripheral vascular disease (RR = 0.90; 95% CI: 0.46 to 1.78), leg amputations (RR = 1.04; 95% CI: 0.44 to 2.44), and microvascular complications (RR = 0.83; 95% CI: 0.59 to 1.17) did not significantly differ between groups (Figure 3). There was no heterogeneity between trials for these end points. The results did not change after sensitivity analyses were performed (see Text S1).

## Discussion

### Major Results

The aim of this meta-analysis was to evaluate the clinical efficacy of metformin in the treatment of T2DM. Surprisingly, this meta-analysis shows no evidence for benefits of metformin in terms of all-cause or cardiovascular mortality and all diabetes macrovascular complications.

Considering the low number of randomised controlled trials included in this meta-analysis and the limited number of events, these results must be interpreted with caution. The consensus recommendations of diabetes experts are that the positive effects of metformin against mortality and CVD observed in UKPDS 34 need confirmation [4],[5]. According to our results, we cannot exclude beyond a reasonable doubt a 25% reduction or a 31% increase in all-cause mortality. We cannot exclude a 33% reduction or a 64% increase cardiovascular mortality.

We used the Mantel–Haenszel odds ratio method with a 0.5 zero-cell correction. This might have somehow biased the results. However, trials with very few or zero events have a very low weight in the meta-analysis. Even though 25 trials met the inclusion criteria, 12 trials could not be included in the meta-analysis because they did not report sufficient information about outcomes of interest.

The observed heterogeneity between studies on the end points mortality and cardiovascular mortality is not totally explained. Trial designs are heterogeneous: follow-up duration (e.g., 4 mo for Garber et al. [22] and Blonde et al. [23], up to 10 y for UKPDS 34 [3]), associated treatments, prior diabetes duration at inclusion, etc. Heterogeneity remained in the subgroup of studies where metformin was not associated with sulphonylurea for the outcome cardiovascular death. Therefore, concomitant treatment with sulphonylurea does not totally explain heterogeneity.

The inclusion of the UKPDS 34(b) subgroup (metformin plus sulphonylurea versus sulphonylurea alone) is what makes our meta-analysis unique. It may partially explain why our results are contradictory with those of previous systematic reviews [6],[7]. The authors of Cochrane systematic reviews excluded this subgroup because their aim was to analyse metformin only as a monotherapy. It is also noteworthy that the international community has emphasised and often cited the favourable results—i.e., showing a benefit from metformin—of UKPDS 34(a), but often not cited the unfavorable results of UKPDS 34(b). However, both groups are randomised and present the same level of evidence. The fact that UKPDS 34(a) is often cited but UKPDS 34(b) is not may be an example of biased knowledge created by excessively citing of a positive result [26]. Lamanna and al. [9] included the UKPDS 34(b) group in their meta-analysis, which had non-diabetic patients and those with type 1 and 2 diabetes, and obtained the same results as we did. Although they put forward the lack of proof for the overall benefit of metformin on cardiovascular events, they concluded that compared to placebo or no treatment, metformin has a benefit. Unlike Lamanna et al., we included different types of control groups (i.e., diet alone, placebo, no treatment) and included add-on therapy and metformin withdrawal studies, and we considered only T2DM. Our conclusion is that the clinical benefit of metformin is far from being demonstrated.

The deleterious effect of the combination of metformin plus sulphonylurea remains unexplained. Five studies in this meta-analysis compared metformin as an add-on therapy in patients receiving sulphonylurea [3],[18],[22]–[24]. There were more deaths, RR = 1.55 (95% CI: 1.03 to 2.33), but this result was mainly related to UKPDS 34 (35.1% of weight). In the ADVANCE study, the combination of sulphonylurea plus metformin was more frequent in the intensive treatment group. No increased risk of mortality was shown [27]. The RECORD study found the combination of metformin plus sulphonylurea “equivalent” to rosiglitazone on both outcomes: all-cause deaths and cardiovascular deaths [28]. However, rosiglitazone was removed from the European market because of safety concerns. Observational studies of metformin combination with sulphonylurea show contradictory results. Two recent studies did not find an increased risk [29],[30], whereas another study [31] and a meta-analysis of observational studies [32] suggest an increased risk of composite end points of CVD, hospital stays, or mortality (fatal and non-fatal events): RR = 1.43 (95% CI: 1.10 to 1.85).

The results of UKPDS 34(a) and (b) may be due to chance alone. Even though it was a randomised study, UKPDS 34 presents methodological weaknesses: the primary end point and study length were modified during the study, after notification of unfavourable results [33]–[36]. The absence of a placebo group and double-blinding could overestimate the benefits of metformin [37],[38]. There may be a bias in the follow-up and assessment of patients or an imbalance of concomitant treatments (such as statins or antihypertensive agents). Details on concomitant treatments received by the study participants in UKPDS 34 have not been published. The authors of the UKPDS 34 10-y follow-up did not provide explanations for and did not discuss the possible toxicity of the combination of metformin and sulphonylurea [39].

Moreover, metformin has no proven efficacy against the occurrence of microvascular complications. The fact that metformin might be ineffective is a possibility that should not be excluded. Metformin belongs to the biguanid class. The first molecule of this class, phenformin, induced increased cardiovascular risk in the University Group Diabetes Program study, which was a double-blind randomised controlled trial versus placebo [12].Pharmacologically speaking, there are few differences between metformin and phenformin [40]. Phenformin is monosubstituted by a longer side chain than metformin, thus conferring lipophilic characteristics and a greater affinity for mitochondrial membranes and an inhibitory effect on the functioning of the mitochondrial respiratory chain. These small molecular differences may explain a decreased risk of lactic acidosis with metformin [40], but are they enough to explain the only favourable results observed in the UKPDS 34(a) subgroup?

Our dataset did not allow a valid evaluation of the benefit of metformin on intermediate end points. Hirst et al. [41] performed a meta-analysis to address this question. Their work supports a clinically important lowering of glycated haemoglobin c (HbA1c) when metformin is used as a monotherapy and in combination with other therapeutic agents.

We were surprised by the small number of studies with enough evidence to evaluate the efficacy of metformin. This is consistent with the findings of Shaughnessy and Slawson [42] and Gandhi et al. [43]. They show that in a sample of registered, ongoing randomised controlled trials on diabetes, only 18% included patient-relevant outcomes as primary outcomes. The vast majority of clinical trials evaluating the efficacy of glucose-lowering drugs in diabetic patients use HbA1c levels as the primary outcome. This is often considered sufficient for licensing. However, because there is a lack of clinical evidence supported by a double-blind randomised controlled trial versus placebo on the clinical efficacy of antidiabetic drugs, it is not possible to prove the ability of HbA1c to predict and capture the effect of treatments [44]. HbA1c cannot be considered as a valid surrogate end point to establish the clinical efficacy of antidiabetic drugs according to the current state of scientific knowledge. In the UKPDS 34(b) subgroup [3], the combination of sulphonylurea and metformin lowered HbA1c levels more than in the group that took only sulphonylurea. The median rate at 4 y was 7.7% versus 8.2%, respectively. However, an excess of mortality was found in the group receiving the combined therapy.

### Policy Implications

Metformin is universally recommended as the first-line treatment for T2DM, even though available evidence of its clinical efficacy is scarce. What should we think about the efficacy of other antidiabetic treatments? Lamanna et al. [9] compared metformin with other hypoglycaemic drugs, and found no difference for cardiovascular end points (OR = 1.03; 95% CI: 0.72 to 1.77, *p* = 0.89). This may be because all treatments have a real clinical benefit that was not demonstrated, or that none of them is beneficial. A large number of patients have taken these treatments over many years, even though there is the possibility of an overall unfavourable benefit/risk ratio. Of note, metformin can induce severe adverse effects such as lactic acidosis in the case of acute renal failure [45] or vitamin B12 deficiency [46].

If doctors doubt the efficacy of metformin because of our results, they may be tempted to prescribe other antidiabetic drugs whose benefits are even less well known. It is not certain whether this is beneficial for patients. In their meta-analysis of retrospective cohort studies, Tzoulaki et al. [47] compared metformin monotherapy with first- or second-generation sulphonylureas on the risk of mortality and congestive heart failure. Their results showed a significant increase (24%–61%) in all-cause mortality associated with first-generation sulphonylureas, while second-generation sulphonylureas were associated with an 18% to 30% increase in congestive heart failure. Insulin therapy is potentially associated with an increase in all-cause mortality [48], especially in patients with heart failure [49]. Sulphonylurea and insulin therapy may be associated with an increase in cancer mortality [50]. In a recent cohort study including more than 62,000 patients, Currie et al. [51] provided evidence that sulphonylurea and insulin treatments in monotherapy are associated with an increased risk of solid cancers (HR = 1.36 and 1.42, respectively) compared to metformin [51]. After a marketing period of more than 10 y, the European Medicines Agency decided to withdraw rosiglitazone from the European market because of its unfavourable benefit/risk ratio, while the US Food and Drug Administration restricted its use. The adverse effects, such as myocardial infarction or death from cardiovascular causes, are well documented [52]. The increased risk of congestive heart failure and weight gain makes the benefit/risk ratio of pioglitazone unclear [53].

Compared with other antidiabetic treatments, metformin may be the one with the least disadvantages. It does not induce hypoglycaemia, weight gain, and heart failure. It is also associated with a reduced rate of mortality among patients with atherothrombosis [54].

### Conclusion

The specific efficacy of metformin to prevent death or cardiovascular events has not been proven by current studies. The number and quality of available studies are insufficient. We cannot exclude beyond any reasonable doubt that metformin use increases or decreases the risk of all-cause mortality or cardiovascular mortality. Further studies are needed to clarify this problematic situation. Metformin may not be the best comparator for evaluating new hypoglycaemic drugs. However, it is not clear which comparator has the most favourable risk/benefit ratio.

## Supporting Information

Text S1 **Appendix and PRISMA checklist.**
(DOC)

Text S2 **Characteristics of studies or subgroups excluded from the meta-analysis.**
(DOC)

## Abbreviations

BMI: body mass index
CVD: cardiovascular disease
HbA1c: glycated haemoglobin c
RR: risk ratio
T2DM: type 2 diabetes mellitus
UKPDS 34: UK Prospective Diabetes Study

## References

[1] Wild S, Green A, Sicree R, King H (2004). Global prevalence of diabetes: estimates for the year 2000 and projections for 2030.. Diabetes Care.

[2] The Emerging Risk Factors Collaboration (2010). Diabetes mellitus, fasting blood glucose concentration, and risk of vascular disease: a collaborative meta-analysis of 102 prospective studies.. Lancet.

[3] UK Prospective Diabetes Study (UKPDS) Group (1998). Effect of Intensive blood-glucose control with metformin on complications in overweight patients with type 2 diabetes (UKPDS 34).. Lancet.

[4] Nathan DM, Buse JB, Davidson MB, Ferrannini E, Holman RR (2009). Medical management of hyperglycemia in type 2 diabetes: a consensus algorithm for the initiation and adjustment of therapy.. Diabetes Care.

[5] National Institute for Health and Clinical Excellence (2008). Type 2 diabetes: the management of type 2 diabetes.. http://www.nice.org.uk/CG66.

[6] Saenz A, Fernandez-Esteban I, Mataix A, Ausejo Segura M, Roqué i Figuls M (2005). Metformin monotherapy for type 2 diabetes mellitus.. Cochrane Database Syst Rev.

[7] Selvin E, Bolen S, Yeh HC, Wiley C, Wilson LM (2008). Cardiovascular outcomes in trials of oral diabetes medications: a systematic review.. Arch Intern Med.

[8] Bennett WL, Maruthur NM, Singh S, Segal JB, Wilson LM (2011). Comparative effectiveness and safety of medications for type 2 diabetes: an update including new drugs and 2-drug combinations.. Ann Intern Med.

[9] Lamanna C, Monami M, Marchionni N, Mannucci E (2011). Effect of metformin on cardiovascular events and mortality: a meta-analysis of randomised clinical trials.. Diabetes Obes Metab.

[10] Rachmani R, Slavachevski I, Levi Z, Zadok B, Kedar Y (2002). Metformin in patients with type 2 diabetes mellitus: reconsideration of traditional contraindications.. Eur J Intern Med.

[11] Cryer D, Mills D, Nicholas SP, Stadel BV, Henry DH (2005). Comparative outcomes study of metformin intervention versus conventional approach.. Diabetes Care.

[12] The University Group Diabetes Program (1975). A study of the effects of hypoglycemic agents on vascular complications in patients with adult-onset diabetes. V. Evaluation of phenformin therapy.. Diabetes.

[13] Jadad AR, Moore RA, Carroll D, Jenkinson C, Reynolds DJ (1996). Assessing the quality of reports on randomised clinical trials: Is blinding necessary?. Control Clin Trials.

[14] Higgins JP, Thompson SG (2002). Quantifying heterogeneity in a meta-analysis.. Stat Med.

[15] Rücker G, Schwarzer G, Carpenter JR, Schumacher M (2008). Undue reliance on I(2) in assessing heterogeneity may mislead.. BMC Med Res Methodol.

[16] Kooy A, De Jager J, Lehert P, Bets D, Wulffelé MG (2009). Long-term effects of metformin on metabolism and microvascular and macrovascular disease in patients with type 2 diabetes mellitus.. Arch Intern Med.

[17] Teupe B, Bergis K (1991). Prospective randomised two-years clinical study comparing additional metformin treatment with reducing diet in type 2 diabetes.. Diabete Metab.

[18] Hermann LS, Scherstén B, Bitzén PO, Kjellström T, Lindgärde F (1994). Therapeutic comparison of metformin and sulfonylurea, alone and in various combinations. A double-blind controlled study.. Diabetes Care.

[19] Horton E, Foley J, Clinkingbeard C, Mallows S, Gatlin M (2000). Nateglinide alone and in combination with Metformin improves glycemic control by reducing mealtime glucose levels in type 2 diabetes.. Diabetes Care.

[20] Hermann LS, Kalén J, Katzman P, Lager I, Nilsson A (2001). Long-term glycaemic improvement after addition of metformin to insulin in insulin-treated obese type 2 diabetes patients.. Diabetes Obes Metab.

[21] Hällsten K, Virtanen KA, Lönnqvist F, Sipilä H, Oksanen A (2002). Rosiglitazone but not metformin enhances insulin- and exercise-stimulated skeletal muscle glucose uptake in patients with newly diagnosed type 2 diabetes.. Diabetes.

[22] Garber AJ, Donovan DS, Dandona P, Bruce S, Park JS (2003). Efficacy of glyburide/metformin tablets compared with initial monotherapy in type 2 diabetes.. J Clin Endocrinol Metab.

[23] Blonde L, Rosenstock J, Mooradian AD, Piper BA, Henry D (2002). Glyburide/metformin combination product is safe and efficacious in patients with type 2 diabetes failing sulphonylureas therapy.. Diabetes Obes Metab.

[24] DeFronzo RA, Goodman AM (1995). Efficacy of metformin in patients with non-insulin-dependent diabetes mellitus. The Multicenter Metformin Study Group.. N Engl J Med.

[25] Chiasson JL, Nadtich L, for the Miglitol Canadian University Investigator Group (2001). The synergistic effect of miglitol plus metformin combination therapy in the treatment of type 2 diabetes.. Diabetes Care.

[26] Greenberg SA (2009). How citation distortions create unfounded authority: analysis of a citation network.. BMJ.

[27] ADVANCE Collaborative Group (2008). Intensive blood glucose control and vascular outcomes in patients with type 2 diabetes.. N Engl J Med.

[28] Home PD, Pocock SJ, Beck-Nielsen H, Curtis PS, Gomis R (2009). Rosiglitazone evaluated for cardiovascular outcomes in oral agent combination therapy for type 2 diabetes (RECORD): a multicentre, randomised, open-label trial.. Lancet.

[29] Azoulay L, Schneider-Lindner V, Dell'aniello S, Schiffrin A, Suissa S (2010). Combination therapy with sulphonylureas and metformin and the prevention of death in type 2 diabetes: a nested case-control study.. Pharmacoepidemiol Drug Saf.

[30] Andersson C, Olesen JB, Hansen PR, Weeke P, Norgaard ML (2010). Metformin treatment is associated with a low risk of mortality in diabetic patients with heart failure: a retrospective nationwide cohort study.. Diabetologia.

[31] Sillars B, Davis WA, Hirsch IB, Davis TM (2010). Sulphonylureas-metformin combination therapy, cardiovascular disease and all-cause mortality: the Fremantle Diabetes Study.. Diabetes Obes Metab.

[32] Rao AD, Kuhadiya N, Reynolds K, Fonseca VA (2008). Is the combination of sufonylureas and metformin associated with an increased risk of cardiovascular disease or all-cause mortality.. Diabetes Care.

[33] Nathan DM (1998). Some answers, more controversy, from UKPDS.. Lancet.

[34] Ewart RM (2001). The case against agressive treatment of type 2 diabetes: critique of the UK prospective diabetes study.. BMJ.

[35] McCormack J, Greenhalgh T (2000). Seeing what you want to see in randomised controlled trials: versions and perversions of UKPDS data.. BMJ.

[36] UK Prospective Diabetes Study (UKPDS) Group (1998). Intensive blood-glucose control with sulphonylureas or insulin compared with conventional treatment and risk of complications in patients with type 2 diabetes (UKPDS 33).. Lancet.

[37] Schulz KF, Chalmers I, Hayes RJ, Altman DG (1995). Empirical evidence of bias. Dimensions of methodological quality associated with estimates of treatment effects in controlled trials.. JAMA.

[38] Moher D, Pham B, Jones A, Cook DJ, Jadad AR (1998). Does quality of reports of randomised trials affect estimates of intervention efficacy reported in meta-analyses?. Lancet.

[39] Holman RR, Matthews DR, Neil HA (2009). Follow-up of intensive glucose control in type 2 diabetes. The authors reply.. N Engl J Med.

[40] Bailey C (1996). Metformin.. N Engl J Med.

[41] Hirst JA, Farmer AJ, Ali R, Roberts NW, Stevens RJ (2012). Quantifying the effect of metformin treatment and dose on glycemic control.. Diabetes Care.

[42] Shaughnessy AF, Slawson DC (2003). What happened to the valid POEMs? A survey of review articles on the treatment of type 2 diabetes.. BMJ.

[43] Gandhi GY, Murad MH, Fujiyoshi A, Mullan RJ, Flynn DN (2008). Patient-important outcomes in registered diabetes trials.. JAMA.

[44] Prentice RL (1989). Surrogate endpoints in clinical trials: definition and operational criteria.. Stat Med.

[45] Fitzgerald E, Mathieu S, Ball A (2009). Metformin associated lactic acidosis.. BMJ.

[46] De Jager J, Kooy A, Lehert P, Wulffelé MG, van der Kolk J (2010). Long term treatment with metformin in patients with type 2 diabetes and risk of vitamin B-12 deficiency: randomised placebo controlled trial.. BMJ.

[47] Tzoulaki I, Molokhia M, Curcin V, Little MP, Millett CJ (2009). Risk of cardiovascular disease and all cause mortality among patients with type 2 diabetes prescribed oral antidiabetes drugs: retrospective cohort study using UK general practice research database.. BMJ.

[48] Gamble JM, Simpson SH, Eurich DT, Majumdar SR, Johnson JA (2010). Insulin use and increased risk of mortality in type 2 diabetes: a cohort study.. Diabetes Obes Metab.

[49] Eurich DT, McAlister FA, Blackburn DF, Majumdar SR, Tsuyuki RT (2007). Benefits and harms of antidiabetic agents in patients with diabetes and heart failure: systematic review.. BMJ.

[50] Bowker SL, Majumdar SR, Veugelers P, Johnson JA (2006). Increased cancer-related mortality for patients with type 2 diabetes who use sulphonylureas or insulin.. Diabetes Care.

[51] Currie CJ, Poole CD, Gale EA (2009). The influence of glucose-lowering therapies on cancer risk in type 2 diabetes.. Diabetologia.

[52] Nissen SE, Wolski K (2007). Effect of rosiglitazone on the risk of myocardial infarction and death from cardiovascular causes.. N Eng J Med.

[53] Richter B, Bandeira-Echtler E, Bergerhoff K, Clar C, Ebrahim SH (2006). Pioglitazone for type 2 diabetes mellitus.. Cochrane Database Syst Rev.

[54] Roussel R, Travert F, Pasquet B, Wilson PWF, Smith SC (2010). Metformin use and mortality among patients with diabetes and atherothrombosis.. Arch Intern Med.

