# Supplementary material for: Reappraisal of Metformin Efficacy in the Treatment of Type 2 Diabetes: A Meta-Analysis of Randomised Controlled Trials
Source: PLoS Med. 2012 Apr 10;9(4):e1001204. doi: 10.1371/journal.pmed.1001204 (PMC3323508; doi:10.1371/journal.pmed.1001204)
Supplement: Text S2 — Characteristics of studies or subgroups excluded from the meta-analysis. (DOC) [file pmed.1001204.s002.doc]

**Text S2 Characteristics of studies or subgroups excluded from the meta-analysis**

| Study  (year of publication) | Randomised (Y/N)  Double-blind / Open | Participants  MET / Control | Treatments | Follow-up  (weeks) | Primary endpoint | Main inclusion criteria |
| --- | --- | --- | --- | --- | --- | --- |
| Dornan 1991 | YES Double-Blind | 30 / 30 | MET /PBO | 32 | NA | HbA1c 5.5-8 BMI 29 |
| Grant 1996, | YES Double-Blind | 25/27/23 analysed 13/14/17 | MET 1500 mg/ MET 3000 mg // PBO | 24 | Metabolic syndrome parameters (PAI1) | FPG >= 6 mmol/l BMI >= 25 |
| Hoffmann 1997, | YES Open | 96  31/32 | MET / PBO | 24 | HbA1c | BMI <= 37 |
| Tamez Pérez 1997b, | YES Open | 20 / 32 | MET / Diet | 12 | FPG  HbA1c | BMI 30 |
| Lee 1998, | YES double Blind | 24 / 24 | MET / PBO | 24 | Body Weight | Women with obesity |
| Damsbo 1998, | J=3  Double-Blind | 18 9/9 | MET / PBO | 12 | Glycogen synthase activity (euglycemic clamp) | BMI > 25 |
| Uehara 2001, | YES Double-Blind | 13/13 analysed 11/11 | MET /PBO | 12 | 24hour blood pressure profile | Diabetic patients with hypertension |
| Mather 2001, | J=2 Double-Blind | 44 29/15 analysed 28/15 | MET / PBO | 12 | Endothelial function | Stable weight, free of Cv disease |
| Hallsten 2002,  also a rosiglitazone group | J=0 NA | 13/14 | MET / PBO | 26 | Muscle sensitivity to insulin | 6.1 < FPG <11.1 BMI 25-40 No complication |
| Del Prato 2003,  also a benfluorex group | J=2 Double-Blind | 284/144 analysed 250/127 | MET / PBO | 24 | HbA1c | patients on diet treatment or low-dose OAD. BMI 25–40 kg/m2 |
| Douek 2005, | J=5 Double-Blind | 183 92 / 91 analysed 87/88 | MET / PBO Insulin initiation | 52 | Weight gain | Patients requiring insulin, with maximum OAD |
| Vähätalo 2007 | YES Open | 11/15/26 | Insulin/Insulin + SU / Insulin + MET | 52 | HBA1c Weight gain | Diabetes > 5years  BMI < 35 HbA1c > 7.5 |

NA: not available MET : Metformin ; SU : Sulfonylureas ; FPG = Fasting Plasma Glucose ; I : Insulinotherapy ; Pbo : Placebo; BMI: Body Mass Index; BW = body weight; OAD oral antidiabetic drug .

**References of Excluded Studies**

Dornan TL, Heller SR, Peck GM, Tattersall RB (1991) Double-blind evaluation of efficacy and tolerability of metformin in NIDDM Diabetes Care 14: 342–4.

Grant PJ (1996) The effects of high- and medium-dose metformin therapy on cardiovascular risk factors in patients with type II diabetes. Diabetes Care 19: 64–6.

Hoffmann J, Spengler M (1997) Efficacy of 24-week monotherapy with acarbose,metformin, or placebo in dietary-treated NIDDM patients: the Essen-II Study. American Journal of Medicine 103: 483– 90.

Tamez Pérez HE, Gómez de Ossio MD, Ibarra Martínez IB (1997) Normoglucemia in newly diagnosed no insulin dependent diabetes mellitus. Non pharmacologic therapy vs. pharmacologic therapy Medicina Interna de Mexico 13: 272–5.

Lee A, Morley JE (1998) Metformin decreases food consumption and induces weight loss in subjects with obesity with type II non-insulindependent diabetes. Obesity Research 6: 47–53.

Damsbo P, Hermann LS, Vaag A, Hother-Nielsen O, Beck-Nielsen H (1998) Irreversibility of the defect in glycogen synthase activity in skeletal muscle from obese patients with NIDDM treated with diet and metformin. Diabetes Care 21: 1489–94.

Uehara MH, Kohlmann NEB, ZanellaMT, Ferreira SRG (2001) Metabolic and haemodynamic effects of metformin in patients with type 2 diabetesmellitus and hypertension. Diabetes Obesity and Metabolism 3: 319–25.

Mather KJ, Verma S, Anderson TJ (2001) Improved endothelial function with metformin in type 2 diabetes mellitus. Journal of the American College of Cardiology 37: 1344–50.

Del Prato S, Erkelens DW, Leutenegger M (2003) Six-month efficacy of benfluorex vs. placebo or metformin in diet-failed type 2 diabetic patients. Acta Diabetologica 40: 20–7.

Douek IF, Allen SE, Ewings P, Gale EA, Bingley PJ; Metformin Trial Group (2005) Continuing metformin when starting insulin in patients with Type 2 diabetes: a double-blind randomized placebo-controlled trial. Diabet Med 22: 634-40.

Vähätalo M, Rönnemaa T, Viikari J (2007) Recognition of fasting or overall hyperglycaemia when starting insulin treatment in patients with type 2 diabetes in general practice. Scand J Prim Health Care 25: 147-53.
